# Lutein, Zeaxanthin and Meso-zeaxanthin Supplementation Associated with Macular Pigment Optical Density

**DOI:** 10.3390/nu8070426

**Published:** 2016-07-12

**Authors:** Le Ma, Rong Liu, Jun Hui Du, Tao Liu, Shan Shan Wu, Xiao Hong Liu

**Affiliations:** 1 The First Affiliated Hospital, Xi’an Jiaotong University College of Medicine, 277 Yanta West Road, Xi’an 710061, Shaanxi, China; 2 School of Public Health, Xi’an Jiaotong University Health Science Center, 76 Yanta West Road, Xi’an 710061, Shaanxi, China; liu.rong@stu.xjtu.edu.cn; 3 The 3201 Hospital, Xi’an Jiao tong University College of Medicine, 783 Tianhan Road, Hanzhong 723000, Shaanxi, China; taoliustone@163.com; 4 The Ninth Hospital of Xi’an, Xi’an Jiaotong University College of Medicine, 151 East of South Second Ring Road, Xi’an 710054, Shaanxi, China; djh79918@163.com; 5 National Clinical Research Center of Digestive Diseases, Beijing Friendship Hospital, Capital Medical University, 95 Yongan Road, Beijing 100050, China

**Keywords:** lutein, zeaxanthin, meso-zeaxanthin, macular pigment optical density

## Abstract

The purpose of this study was to evaluate the effects of lutein, zeaxanthin and meso-zeaxanthin on macular pigment optical density (MPOD) in randomized controlled trials (RCTs) among patients with age-related macular degeneration (AMD) and healthy subjects. Medline, Embase, Web of Science and Cochrane Library databases was searched through May 2016. Meta-analysis was conducted to obtain adjusted weighted mean differences (WMD) for intervention-versus-placebo group about the change of MPOD between baseline and terminal point. Pearson correlation analysis was used to determine the relationship between the changes in MPOD and blood xanthophyll carotenoids or baseline MPOD levels. Twenty RCTs involving 938 AMD patients and 826 healthy subjects were identified. Xanthophyll carotenoids supplementation was associated with significant increase in MPOD in AMD patients (WMD, 0.07; 95% CI, 0.03 to 0.11) and healthy subjects (WMD, 0.09; 95% CI, 0.05 to 0.14). Stratified analysis showed a greater increase in MPOD among trials supplemented and combined with meso-zeaxanthin. Additionally, the changes in MPOD were related with baseline MPOD levels (*r*_AMD_ = −0.43, *p* = 0.06; *r*_healthy subjects_ = −0.71, *p* < 0.001) and blood xanthophyll carotenoids concentration (*r*_AMD_ = 0.40, *p* = 0.07; *r*_healthy subjects_ = 0.33, *p* = 0.05). This meta-analysis revealed that lutein, zeaxanthin and meso-zeaxanthin supplementation improved MPOD both in AMD patients and healthy subjects with a dose-response relationship.

## 1. Introduction

The macula is a specialized part in the posterior pole of retina, since it mediates central vision, provides the sharpest visual acuity and facilitates the best color discrimination [1]. As the major functional component in the macular region, macular pigment (MP) was uniquely concentrated in the inner and central layers and mainly composed of xanthophyll carotenoids, including lutein, zeaxanthin and meso-zeaxanthin [2,3,4,5,6,7,8]. The concentration of these carotenoids in the macular region is about 1000 times greater than that in the blood [8]. The exquisite degree of biological selectivity in the retina indicated that these carotenoids played a pivotal role in maintaining the normal morphology and function of the macula [9]. Furthermore, lutein, zeaxanthin and meso-zeaxanthin are believed to play a major role in protecting retina and retinal pigment epithelium from light-initiated oxidative damage by scavenging reactive oxygen species and filtering blue light, which was involved in the putative pathogenesis of many age-related eye diseases [10,11,12,13,14,15]. Thus, elevated MP affords protection against the development of many retinal diseases, especially for age-related macular degeneration (AMD); contrarily, low MP enhanced the risk of these diseases [4,6,12,13].

Data from epidemiologic studies suggested that dietary lutein and zeaxanthin intake were inversely associated with the risk of AMD [16,17,18]. In addition, our previous studies also found that supplementation with these macular carotenoids partially reversed the loss of visual function in patients with early AMD by elevating macular pigment optical density (MPOD), suggesting a causative role of MPOD for the maintenance of normal visual function [19]. Although some intervention studies have showed that lutein, zeaxanthin and meso-zeaxanthin supplementation resulted in significant morphologic changes in macular pigment, the response was variable among different studies and even a few studies failed to find such an increase in MPOD [20,21,22]. Populations with specific genetic backgrounds or nutritional status may potentially affect the transport and deposition processes of these carotenoids from blood to macula during supplementation [13,17]. The efficacy of supplementation for the different study populations and supplement dose remained uncertain. Furthermore, total zeaxanthin increases with decreasing eccentricity in the macula, and tends to be the dominant carotenoid at the central fovea [23]. These specific distribution patterns suggest that zeaxanthin may play a crucial role in the center of the retina. In addition, It was hypothesized that meso-zeaxanthin, a geometrical isomer of zeaxanthin, was able to protect against age-related eye damage by the special antioxidant properties and light filtering properties [5,24,25]. However, whether zeaxanthin and meso-zeaxanthin should be added in combination with lutein remained to be confirmed. Besides, MPOD depends on the stimuli that are used for its measurement [19,21]. Thus, the influence of different methods used in included studies should be explored.

Therefore, we performed a meta-analysis of randomized controlled trials (RCTs) to determine the effect of lutein, zeaxanthin and meso-zeaxanthin supplementation on MPOD in AMD patients and healthy subjects.

## 2. Materials and Methods

This meta-analysis was conducted according to the Preferred Reporting Items for Systematic Reviews and Meta-Analyses (PRISMA) guidelines [26].

### 2.1. Data Sources and Search Strategy

A comprehensive search was performed to identify all relevant articles in Medline, Embase, Web of Science, and Cochrane Library database up to May 2016, using the search terms lutein, zeaxanthin, meso-zeaxanthin, xanthophyll or carotenoids in conjunction with each of the following words: macular pigment optical density, macular pigment density, macular pigment, MPOD and MP, as well as combinations of these terms. References from retrieved articles were also reviewed for pertinent studies. No language restriction was applied for searching and study inclusion. Experts in the field were content in terms of additional information or potential unpublished studies in the case of missing data.

### 2.2. Study Selection

The titles and abstracts of potentially eligible studies were identified by the search strategy. Then, the full text articles were reviewed to determine whether they met the inclusion criteria. Studies were included in the meta-analysis if they fulfilled the following criteria: (1) eligible studies were limited to randomized controlled trials (RCTs); (2) subjects were randomized to receive lutein, zeaxanthin or/and meso-zeaxanthin supplement or placebo; (3) the outcome of interest was MPOD; (4) studies reported the change of MPOD between baseline and at the end of study in the intervention and placebo group. When studies were conducted in healthy subjects, these subjects should be free of retinal disease. If multiple articles were published from the same study, only the most updated data was selected for analysis. Three investigators (Rong Liu, Jun Hui Du and Tao Liu) independently reviewed all identified publications for inclusion using predetermined criteria, with discrepancies resolved by consensus.

### 2.3. Data Extraction and Study Quality Assessment

For each included study, study characteristics and demographics was recorded as follows: first author, publication year, sample size, population characteristics (age, sex and country), interventions (dose of lutein/zeaxanthin/meso-zeaxanthin and duration of follow-up), change in the mean with standard deviation (SD) for MPOD, numbers enrolled and lost to follow-up. This needs to be clear in the manuscript. When several means and standard deviations were present in a single study, the data was pooled by combining groups into a single group according to the Cochrane recommendation. Where final SDs were not available from trials, they were calculated from confidence intervals (CI) or standard errors reported in study. If the information of blood lutein and zeaxanthin concentration was showed in studies, it was also extracted for further relevant analysis.

Methodological quality of each study was evaluated by the Jadad score, a 5-point study quality assessment instrument. This scale consists of three aspects: the method of randomization, the adequacy of blinding, and the description of withdrawals and dropouts. Studies that scored three or more were considered to be categorized as high quality. Data extraction and quality assessment was conducted independently and in duplicate by three investigators (Rong Liu, Jun Hui Du and Tao Liu), and any disagreement was adjudicated by a fourth author (Le Ma).

### 2.4. Statistical Analysis

The weighted mean differences (WMD) and corresponding 95% CIs were used as the primary summary measure of the effect of lutein/zeaxanthin/meso-zeaxanthin supplement on MPOD. Statistical heterogeneity among studies was evaluated by Q tests and the degree of heterogeneity was assessed by I^2^ statistics. WMD for MPOD were pooled using inverse-variance weighting with the fixed effects or random-effects models. To explore the potential sources of between-study heterogeneity, meta-regression analyses were conducted stratified by health status (AMD patients vs. healthy participants), dose of lutein, zeaxanthin or meso-zeaxanthin supplementation (>10 mg vs. ≤10 mg), duration of intervention (≥12 month vs. <12 month), mean age of subjects (>70 years vs. ≤70 years), zeaxanthin (with zeaxanthin vs. without zeaxanthin), meso-zeaxanthin (with meso-zeaxanthin vs. without meso-zeaxanthin ), other antioxidants use (with other antioxidants vs. without other antioxidants) and geographic area (Europe vs. Asia vs. North America), measurement method of MPOD (objective (fundus autofluorescence, spectral fundus reflectance and VISUCAM NM/FA) vs. psychophysical (heterochromatic flicker photometry and macular assessment profile)) [27]. In pooling dose-response analysis, the relationship between the dose of lutein/zeaxanthin/meso-zeaxanthin supplement and the change in MPOD in each study was examined by linear regression model. The association between the increase in MPOD and blood xanthophyll carotenoids concentration was investigated using Pearson correlation analysis. Sensitivity analyses to examine the influence of each individual study were performed by iteratively excluding each study from this meta-analysis and comparing the point estimates without and with one study at a time. Publication bias was assessed by the Egger regression asymmetry test and the Begg adjusted rank correlation test [28,29]. All statistical analyses were conducted by Stata software, version 10.0 (Stata Corp, College Station, TX, USA). *p* < 0.05 was considered statistically significant.

## 3. Results

### 3.1. Literature Search

A total of 2456 potentially relevant publications were retrieved during our initial search. After duplicate publications detection and abstract review, full-text versions of the remaining 133 articles were then retrieved for detailed evaluation. Of these, 114 retrieved trials were not eligible due to duplicate publications, lack of a control group, outcomes not suitable for the meta-analysis, means or SDs of pretest and posttest data not included in the publication and not provided by the authors on request. Finally, the remaining 20 articles were eligible for inclusion in our analysis [17,20,21,22,30,31,32,33,34,35,36,37,38,39,40,41,42,43,44,45].

### 3.2. Study Characteristics

The characteristics of the included studies are presented in Table 1. In these trials, 12 were performed in Europe, 6 in USA and 2 in China. The number of participants in each study ranged from 19 to 172, comprising a total of 1764. Most studies included both men and women, except for 2 in which only men or women were selected. 8 trials supplemented with lutein vs. placebo, 2 treated with zeaxanthin vs. placebo, 8 intervened by combining lutein and zeaxanthin vs. placebo, and 8 had multiple arms (lutein, zeaxanthin or/and meso-zeaxanthin combined with other antioxidants, vs. placebo). The dosage of lutein, zeaxanthin or/and meso-zeaxanthin in the intervention groups among trials varied from 0 mg/day to 20 mg/day. The duration of intervention and follow-up ranged from 8 weeks to 2 years. MPOD was measured by the objective methods in 7 studies, and psychophysical methods in 13 trials. All included studies had a Jadad score of 3 or more, indicating generally high methodological quality.

### 3.3. The Effect of Lutein, Zeaxanthin or/and Meso-zeaxanthin Supplementation on MPOD in Patients with AMD

**Figure 1:**
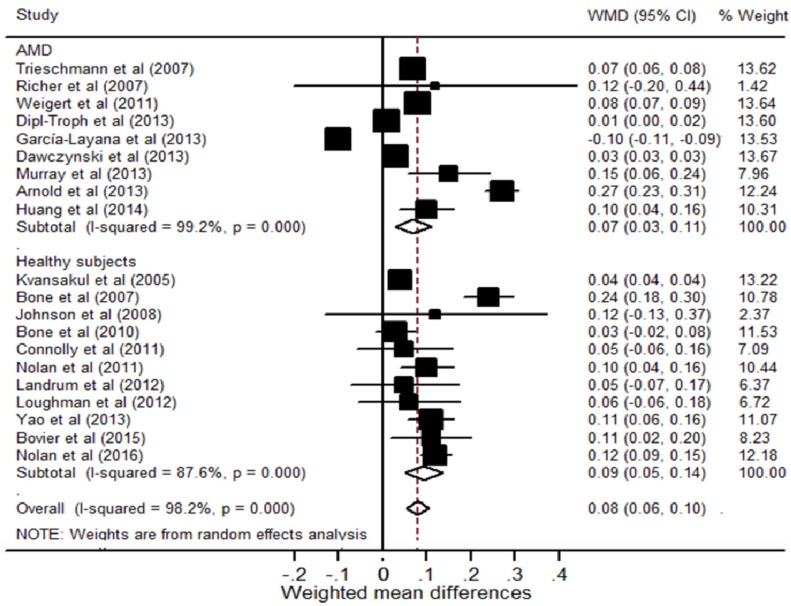
Forest plot showing the efficacy of lutein, zeaxanthin and meso-zeaxanthin supplementation on macular pigment optical density for patients with AMD and healthy subjects. Error bars indicate 95% CIs of the WMDs. The sizes of the squares correspond to the study weight in the random-effects meta-analysis. Diamonds represent the meta-analysis summary effect estimate. AMD, age-related macular degeneration; CI, confidence interval; WMD, weighted mean differences.

Nine RCTs evaluated the efficacy of these carotenoids supplement on the changes in MPOD for AMD patients (Figure 1). The *I*^2^ test for heterogeneity was 99.2% (*p* < 0.001); and the results from random-effects models suggested that combing trials produced a MPOD increase by 0.07 ODU (95% CI, 0.03 to 0.11) in favor of supplementation vs. placebo. In the stratified analysis, a longer supplementation time had a marginally greater effect in comparison with the shorter time (0.17 vs. 0.05; between-group difference, 0.12; *p* = 0.05; Table 2). Trials measured MPOD with objective methods showed a larger increase in MPOD compared with those by psychophysical methods, although the difference did not reach statistical significance (0.09 vs. 0.05; between-group difference, 0.04; *p* = 0.37). The dose-response meta-analysis estimate showed a 0.005 ODU improvement in MPOD for a 1 mg/day increase in these carotenoids supplement. In sensitivity analysis, exclusion of any single trial from the analysis did not alter the overall findings of the effect of supplementation on MPOD. No evidence of publication bias was detected in this study by either Begg (*p* = 0.68) or Egger test (*p* = 0.83).

### 3.4. The Effect of Lutein, Zeaxanthin or/and Meso-zeaxanthin Supplementation on MPOD in Healthy Subjects

The changes in MPOD with these carotenoids supplement for healthy subjects were assessed in 11 RCTs (Figure 1). When all these studies were pooled into the meta-analysis, the intervention group evidently exhibited an augmentation in MPOD by 0.09 ODU compared with placebo (95% CI, 0.05 to 0.14). For subgroup analysis, trials that intervened exceeding 10 mg macular carotenoids per day produced a higher WMD of 0.12 (95% CI, 0.09 to 0.15) than a WMD of 0.05 (95% CI, 0.03 to 0.07) in trials that only supplemented with less than 10 mg (between-group difference, 0.07; *p* = 0.01). Moreover, a greater increase in MPOD was observed in trials supplemented combined with meso-zeaxanthin in comparison with those without meso-zeaxanthin (WMD, 0.13 vs. 0.07; between-group difference, 0.06; *p* = 0.02; Table 2). Additionally, participants receiving additional zeaxanthin supplement did not have a more response in MPOD compared with those who taking only lutein supplement. In the dose-response meta-analysis, each additional 1 mg of these carotenoids supplementation was associated with a 0.004 ODU increase in MPOD. The sensitivity analysis by excluding each of the studies also did not appreciably influence the pooled WMD. No publication bias was found for Begg’s rank correlation test (*p* = 0.54) or Egger’s linear regression test (*p* = 0.05).

### 3.5. The Relationship between Baseline MPOD Levels and the Change in MPOD

**Figure 2:**
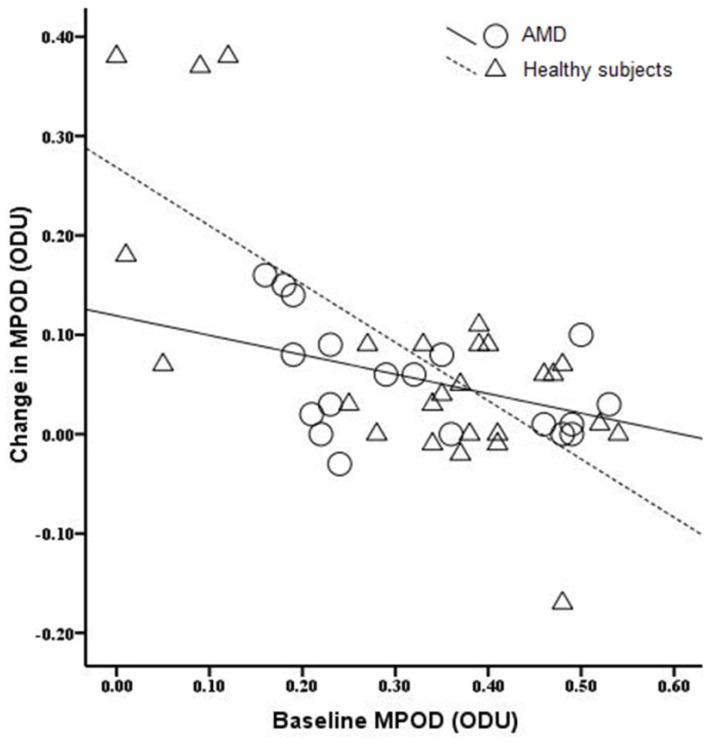
Scatterplot showing the relationship between baseline MPOD levels and the change in MPOD from baseline. MPOD, macular pigment optical density; ODU, optical density unit.

Correlation analysis was used to investigate the association between baseline MPOD levels and the change in MPOD during treatment (Figure 2). For healthy subjects, the changes in MPOD during supplementation were significantly related with baseline levels (*r* = −0.71, *p* < 0.001). Moreover, the increase in MPOD for AMD patients also marginally exhibited a negative correlation with baseline MPOD (*r* = −0.43, *p* = 0.06).

### 3.6. The Relationship between Blood Xanthophyll Carotenoids Concentration and the Change in MPOD

**Figure 3:**
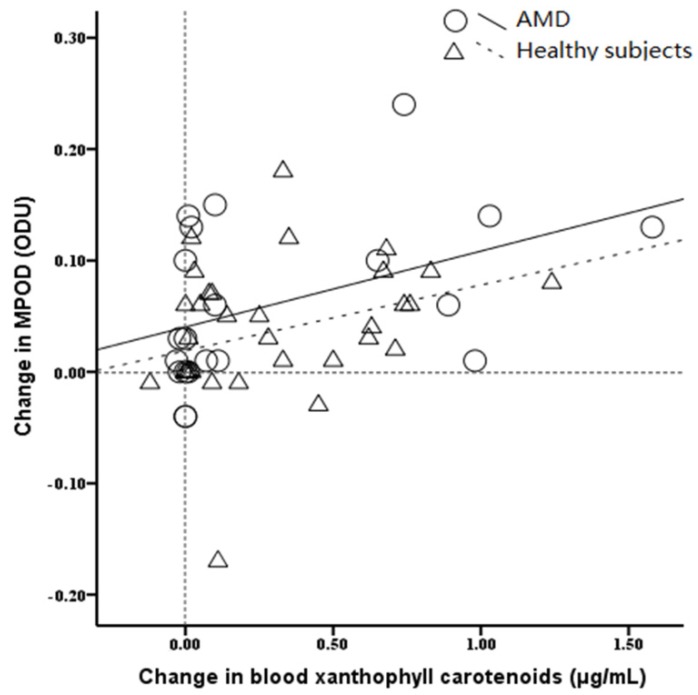
Scatterplot showing the relationship between blood xanthophyll carotenoids concentration and the change in MPOD during supplementation. MPOD, macular pigment optical density; ODU, optical density unit.

We subsequently evaluated the relationship between the change in serum carotenoids concentration and the change in MPOD (Figure 3). The results showed that MPOD was improved with the postintervention increase in blood concentrations both in AMD patients (*r* = 0.40, *p* = 0.07) and the healthy populations (*r* = 0.33, *p* = 0.05).

## 4. Discussion

In the current study, we evaluated the effects of lutein, zeaxanthin and meso-zeaxanthin supplementation on MOPD based on the data from the RCTs. Our results showed that the carotenoids supplementation significantly increased the level of MPOD and the inclusion of meso-zeaxanthin resulted in a greater increase in macular pigment compared to supplements lacking this central carotenoid. The increment in MPOD was positively correlated with changes in blood xanthophyll carotenoids concentration. Furthermore, supplementation with these carotenoids for longer than 12 months, a higher dose and the three carotenoids in combination were more effective on MPOD augmentation.

Previous studies have found that the decrease in MP was related with the functional abnormalities of the macula, which eventually led to some age-related degenerative eye diseases [46,47]. Neuringer et al. reported that monkeys fed with the xanthophyll-free diets were found to have no detectable MP in the retina and adipose tissue [47]. As the main constituents of the yellow pigment, lutein, zeaxanthin and meso-zeaxanthin are uniquely concentrated in the macula [12,48,49]. It is hypothesized that these carotenoids could protect the photoreceptor outer segments and the retinal pigment epithelium by screening these susceptible retinal structures from actinic blue light and quenching reactive oxygen species [50]. Barker et al. demonstrated that lutein and zeaxanthin supplementation of xanthophyll-free monkeys and the resulting accumulation of MP provided significant foveal protection against short-wavelength photochemical damage [11]. Their results were in agreement with those reported by Thomson et al., in which quails supplemented with 6-month xanthophyll carotenoids significantly decreased number of dying photoreceptors in retina [51]. Moreover, these carotenoids have also been suggested to offer protection to reduce the lipofuscin accumulation and enhance in lysosomal stability and viability [52]. Thus, lutein, zeaxanthin and meso-zeaxanthin may have a possible specific function in the maintenance of human retinal structures [7,17,48]. 

Some reports revealed that the donor eyes with AMD showed a drastic decline of MP levels as compared to eyes without AMD [53]. According to previous studies, a lower MPOD appeared to be associated with an increased risk of progression to AMD [54,55]. Our previous intervention study has demonstrated a significant benefit of lutein and zeaxanthin supplementation on the increase of MPOD for patients with early AMD [19]. Consistent with these findings, the results of the present study showed that supplementation with these carotenoids significantly increased the level of MPOD not only in AMD patients but also in healthy subjects. Moreover, the change in MPOD was accompanied by the improvement of these xanthophyll carotenoids statuses. These suggested that supplementation with lutein, zeaxanthin and meso-zeaxanthin lead to the improvements in MPOD as a consequence of maintaining the normal morphology of retina by elevating blood levels [54]. In addition, our results also showed that participants receiving with higher doses supplement were associated with a greater increase in MPOD, especially for the healthy subjects. Previous studies suggested that a consumption of lutein and zeaxanthin above 6-14 mg daily was considered to reduce the risk of eye diseases such as AMD as well as in alleviating the symptoms if present [56,57]. However, epidemiological studies indicated that the combined daily dietary intake of these carotenoids was only approximately 2 mg per day in western countries [58]. Therefore, the additional consumption of these carotenoids supplements should be warranted.

Although zeaxanthin is deposited throughout the human retina, it is preferentially accumulated at the fovea region of macula [59]. Such a specific distribution pattern of these carotenoids within the human macula indicated that combined zeaxanthin and lutein might result in greater improvements in MPOD than lutein alone; however, absence of significantly greater response was noted with combination treatment in the present study. This finding may be partly attributed to the fact that zeaxanthin deposition at the fovea during supplementation may be limited [60,61]. Due to the high chemical similarity of lutein and zeaxanthin, tissue-specific xanthophyll binding proteins may mediate lutein and zeaxanthin capture by competition for the same absorption mediator [61]. Once these protein receptors are saturated, they could not capture more macular xanthophylls, which may limit the amount of zeaxanthin being additionally accumulated [62]. Meanwhile, the relatively higher levels of zeaxanthin naturally present at the central fovea may also limit deposition of zeaxanthin in this area [63]. This hypothesis was also supported by our results that a significant negative association was detected between the changes in MPOD and the baseline levels. Thus, the populations with lower MP may benefit more from the additionally supplementation of xanthophyll carotenoids. Furthermore, meso-zeaxanthin is a different molecular to lutein and zeaxanthin which resides directly over the central of the macula. Although trace amount of meso-zeaxanthin existed in some kind of fish, it could not be found in raw fruits and vegetables, or detected in blood serum [64]. It has the ability to protect against chronic and cumulative eye damage through its capacity to filter the most energetic and potentially damaging wavelengths of visible light and to neutralize free radicals produced by oxidative stress [65]. It has been shown that 1:1:1 mixture of lutein, zeaxanthin and meso-zeaxanthin could quench singlet oxygen more efficiently than any of the three individually. The reason could be explained that three carotenoids may form specific aggregates, which could enhance their ability to quench singlet oxygen [7,17]. Loughman et al. reported the observed change in MPOD was not statistically significant among subjects receiving lutein and zeaxanthin supplementation for 6 months, as the supplement did not contain meso-zeaxanthin [22]. The results of this meta-analysis also indicated that having meso-zeaxanthin in the supplement offers a greater increase in MPOD than supplements lacking this carotenoid, which was in accordance with previous study. In addition, Thurnham and Xu demonstrated that meso-zeaxanthin supplementation caused no noticeable toxicological effects on rats [5,25]. Therefore, additional meso-zeaxanthin supplementation should be encouraged.

Several potential limitations should be taken into account. First, these included studies selected different methods for MPOD measurement. Although the results of the stratified analysis revealed that this factor did not significantly alter the effect of lutein, zeaxanthin or/and meso-zeaxanthin supplementation on MPOD, the potential influence from this factor could not be ruled out completely. As the stimuli that are used for MPOD measurement, such as peak wavelength, width of the measuring and reference lights, stimulus size, varied across studies, our results might also be affected by these potential confounding factors. Second, majority of the studies intervened less than 2 years, and it is unclear whether a higher dosing strategy over time may be associated with greater benefit. Fortunately, the Central Retinal Enrichment Supplementation Trials (CREST) will illustrate the role of longer-term nutritional supplementation in maintaining the levels of xanthophyll carotenoids in blood and macula, and clarify the effects of lutein, zeaxanthin and meso-zeaxanthin on visual function in normal subjects and in subjects with early AMD [66]. Third, the relatively small sample sizes of the included RCTs in this meta-analysis would reduce the statistical power to assess the association between supplementation with the macular carotenoids and MPOD. However, all of the included studies were considered of high quality, which might enhance the reliability of results. Fourth, other variables, like glare disability and dietary supplementation with carotenoid rich foods, are not included in present study. Thus, further research is needed to study the association between different responses and dietary supplementation with carotenoids-rich foods. Finally, although no significant publication bias was detected, the potential bias could not be ruled out.

## 5. Conclusions

The present meta-analysis demonstrated significant benefits of lutein, zeaxanthin and meso-zeaxanthin supplementation on MPOD augmentation both in AMD patients and healthy subjects with a dose-response relationship. Moreover, such improvement was positively associated with the increase in blood xanthophyll carotenoids level. As most of the studies involved less than 12 months of follow-up, which limits the evaluation of extended effect of these carotenoids, further larger-scale and longer-term RCTs are required to examine the effects of xanthophyll carotenoids on protecting the morphological integrity of the retina and preventing the progression of AMD.

## Figures and Tables

**Table 1:** Characteristics of the eligible randomized clinical trials.

Authors (Year)	Study Participants	Trial Duration	No. of Groups	Lnterventions	Measurement Method for MPOD	Follow-Up Rates (%)	Quality Score *
Trieschmann et al. (2007) [20]	130 AMD patients aged (71.4 ± 7.6) years in Germany	6 months	2	12 mg lutein and 1 mg zeaxanthin combined with other antioxidants; placebo	Fundus autofluorescence	94.6	3
Richer et al. (2007) [21]	90 AMD patients aged (74.1 ± 7.5) years in the USA	12 months	3	10 mg lutein; 10 mg lutein combined with other antioxidants; placebo	HFP	84.4	5
Weigert et al. (2011) [30]	126 AMD patients aged (71.6 ± 8.6) years in Austria	6 months	2	20 mg lutein daily in months 1 to 3 and 10 mg lutein daily in months 4 to 6; placebo	Spectral fundus reflectance	87.3	3
Arnold C et al. (2013) [31]	20 AMD patients aged (66.0 ± 8.0) years in Germany	10 weeks	2	10 mg lutein plus 3 mg zeaxanthin; placebo	VISUCAM NM/FA	100.0	5
García-Layana et al. (2013) [32]	44 AMD patients aged (68.5 ± 8.5) years in Spain	12 months	2	12 mg lutein plus 0.6 mg zeaxanthin combined with other antioxidants; placebo	HFP	NR	3
Dawczynski et al. (2013) [33]	172 AMD patients aged (70.0 ± 10.0) years in Germany	12 months	3	10 mg lutein, 1 mg zeaxanthin combined with other antioxidants; 20 mg lutein, 2 mg zeaxanthin combined with other antioxidants; placebo	VISUCAM NM/FA	84.3	3
Murray et al. (2013) [34]	72 AMD patients aged (70.5 ± 8.7) years in UK	12 months	2	10 mg lutein daily; placebo	HFP	86.9	5
Arnold C et al. (2013) [35]	172 AMD patients aged (69.0 ± 10.0) years in Germany	12 months	3	10 mg lutein plus 1 mg zeaxanthin combined with other antioxidants; 20 mg lutein plus 2 mg zeaxanthin combined with other antioxidants; placebo	VISUCAM NM/FA	84.3	5
Huang et al. (2015) [36]	112 AMD patients aged (69.1 ± 7.4) years in China	24 months	4	10 mg lutein; 20 mg lutein; 10 mg lutein plus 10 mg zeaxanthin; placebo	Fundus autofluorescence	96.4	5
Kvansakul et al. (2005) [37]	92 healthy men in UK	12 months	4	10 mg lutein; 10 mg zeaxanthin; 10 mg lutein plus 10 mg zeaxanthin in months 1 to 6 and 20 mg lutein; 20 mg zeaxanthin; 10 mg lutein plus 10 mg zeaxanthin in months 7 to 12; placebo	MAP	79.3	4
Bone et al. (2007) [38]	19 healthy subjects in the USA	120 days	2	14.9 mg of meso-zeaxanthin, 5.5 mg of lutein, and 1.4 mg of zeaxanthin; placebo	HFP	NR	3
Johnson et al. (2008) [39]	57 healthy women in the USA	4 months	3	12 mg lutein plus 0.5 mg zeaxanthin;12 mg lutein plus 800 mg DHA; placebo	HFP	86.0	4
Bone et al. (2010) [40]	100 healthy subjects in the USA	140 days	4	5 mg lutein; 10 mg lutein; 20 mg lutein; placebo	HFP	87.0	4
Connolly et al. (2011) [17]	44 healthy subjects in Ireland	6 months	2	10.6 mg meso-zeaxanthin, 5.9 mg lutein, and 1.2 mg zeaxanthin; placebo	HFP	79.5	5
Nolan et al. (2011) [41]	121 healthy subjects in Ireland	12 months	2	12 mg lutein, 1 mg zeaxanthin combined with other antioxidants; placebo	HFP	62.8	4
Landrum et al. (2012) [42]	30 healthy subjects in the USA	24 weeks	3	20 mg lutein diacetate; 20 mg lutein; placebo	HFP	NR	3
Loughman et al. (2012) [22]	36 healthy subjects in Ireland	6 months	3	20 mg lutein plus 2 mg zeaxanthin; 10 mg meso-zeaxanthin, 10 mg lutein plus 2 mg zeaxanthin; placebo	HFP	88.9	5
Yao et al. (2013) [43]	120 healthy subjects in China	12 months	2	20 mg lutein; placebo	HFP	82.5	4
Bovier et al. (2015) [44]	102 healthy subjects in the USA	4 months	3	20 mg zeaxanthin; 8 mg lutein plus 26 mg zeaxanthin combined with other antioxidants; placebo	HFP	67.6	4
Nolan et al. (2016) [45]	105 healthy subjects in Ireland	12 months	2	10 mg lutein, 2 mg zeaxanthin, and 10 mg meso-zeaxanthin; placebo	Autofluorescence	80.0	5

Abbreviations: AMD, age-related macular degeneration; HFP, heterochromatic flicker photometry; MPOD, macular pigment optical density; NR, not report. * Study quality was judged based on the Jadad scale.

**Table 2:** Stratified analysis for the lutein or/and zeaxanthin or/and meso-zeaxanthin supplements effect on macular pigment optical density (MPOD) across the assessed randomized controlled trials (RCTs).

Subgroup	AMD Patients					Healthy Populations				
	*N*	WMD	95% CI	P_z_	P_h_	*N*	WMD	95% CI	P_z_	P_h_
Dose of supplement										
>10 mg	10	0.07	0.04, 0.12	<0.001	0.93	15	0.12	0.09, 0.15	<0.001	0.01
≤10 mg	4	0.09	−0.07, 0.19	0.40		4	0.05	0.03, 0.07	0.02	
Duration of intervention										
≥12 months	11	0.17	0.09, 0.24	<0.001	0.05	6	0.07	0.04, 0.10	<0.001	0.83
<12 months	3	0.05	0.01, 0.09	<0.001		13	0.08	0.03, 0.13	<0.001	
Mean age										
>70 years	7	0.06	0.03, 0.09	<0.001	0.85					
≤70 years	7	0.11	0.02, 0.19	<0.001						
Zeaxanthin										
With	9	0.07	0.04, 0.11	<0.001	0.60	11	0.09	0.06, 0.13	<0.001	0.21
Without	5	0.08	0.07, 0.09	0.41		8	0.08	0.03, 0.08	0.03	
Meso-zeaxanthin										
With						4	0.13	0.05, 0.22	0.001	0.02
Without						15	0.06	0.03, 0.08	<0.001	
Other antioxidants										
With	7	0.08	0.04, 0.13	<0.001	0.97	3	0.10	0.05, 0.15	0.99	0.55
Without	7	0.08	0.04, 0.13	<0.001		16	0.07	0.05, 0.10	<0.001	
Geographic area										
Europe	9	0.08	0.04, 0.11	<0.001	0.80	8	0.06	0.03, 0.09	<0.001	0.50
Asia	3	0.10	0.05, 0.15	0.27		1	0.11	0.06, 0.16	-	
USA	2	0.12	−0.15, 0.38	0.97		10	0.09	0.02, 0.15	<0.001	
Methods										
Objective	10	0.09	0.07, 0.12	<0.001	0.37					
Psychophysical	4	0.05	−0.15, 0.24	<0.001						

Abbreviations: AMD, age-related macular degeneration; CI, confidence interval; MPOD, macular pigment optical density; P_h_, P for between-study heterogeneity; P_z_, P for Z test; RCTs: randomized controlled trials; WMD, weighted mean differences.
